# The relationship between the sources of confidence in overcoming COVID-19 and the improvement of medical students' professional identity: mediation by medical students' attention to COVID-19

**DOI:** 10.1186/s12909-022-03994-4

**Published:** 2023-01-14

**Authors:** Na Zhu, Zhiyuan Zhang, Jun Xie, Yangli Ou, Jia Tan, Hong Gao

**Affiliations:** 1 School of Nursing, University of South China, Hengyang, China; 2 Emergency Department, The Second Affiliated Hospital, Hengyang Medical School, University of South China, Hengyang, China; 3 Department of Student Affairs, University of South China, Hengyang, China; 4 Department of Neonatology, The First Affiliated Hospital, Hengyang Medical School, University of South China, Hengyang, China; 5 Department of Nursing, The Second Affiliated Hospital, Hengyang Medical School, University of South China, Hengyang, China

**Keywords:** COVID—19, Medical education, Social media, Confidence in overcoming public health crisis, Professional identity

## Abstract

**Background:**

The prevalence of COVID-19 highlights the shortage of human medical resources, and improving medical students' professional identity is crucial to improving this situation. The sources of confidence in overcoming COVID-19 and medical students' attention to COVID-19 were significant factors affecting their professional identity. However, no study has investigated the mediating role of medical students' attention to COVID-19 in their relationship. This study investigates the relationship between these three factors in three medical university students in Hunan Province.

**Methods:**

A cross-sectional survey study that used convenience sampling method was conducted on 2775 medical students from three universities in the Hunan Province of China from March 15 to April 19, 2020. An intermediary model was established to evaluate the role of medical students' attention to COVID-19 in the sources of confidence in overcoming COVID-19 and the improvement of medical students' professional identity.

**Results:**

The sources of confidence in overcoming COVID-19, medical students' attention to national crisis events, and the improvement of medical students' professional identity was positively associated with each other (*β* = 0.328 ~ 0.464, *P* < 0.001). The mediating effect accounted for 23.3% of the total effect and 30.4% of the direct effect. Medical students' attention to COVID-19 partially mediates the relationship between the sources of confidence to overcome COVID-19 and the improvement of medical students' professional identity.

**Conclusions:**

This study found that the sources of confidence in overcoming COVID-19 and medical students' attention to national crisis events have a significant predictive effect on the improvement of medical students' professional identity. Medical students' attention to COVID-19 mediated the relationship between the sources of confidence to overcome COVID-19 and the improvement of medical students' professional identity. The findings have emphasized the theoretical and practical significance of professional identity education for medical students.

## Introduction

The high morbidity and mortality rates of COVID-19 have led to an acute shortage of human medical resources worldwide, which has placed a burden on healthcare systems [1, 2]. Medical students are the reserve army of human medical resources, and improving their professional identity is a critical factor in maintaining the stability of the medical personnel teams. So far, there are few studies on improving medical students' professional identity, especially during major public health events such as the outbreak of COVID-19.

Medical workers are the leading force in the fight against the COVID-19 epidemic, and the government and social workers have provided strong support and cooperation for them to promote the orderly implementation of COVID-19 prevention and control to control the development of the epidemic effectively. The achievements in the prevention and control of COVID-19 include the improvement of the medical security system, the high popularity rate of epidemic prevention and control knowledge, the timely rescue of patients, and the orderly return of enterprises [3, 4]. They are essential sources of confidence for medical students that the whole country can overcome the COVID-19 epidemic. Studies have shown that the more government and society support medical personnel and their work, the stronger the medical student’s professional identity and employment intention [5]. This implies an association between the sources of confidence in overcoming COVID-19 and the improvement of professional identity, but it needs further verification. The underlying mechanism of how the sources of confidence in overcoming COVID-19 are related to improving professional identity among medical students needs to be further explored.

The outbreak of COVID-19 has spread rapidly across the country and attracted extensive attention from society. Social media plays a vital role in the information dissemination and public opinion guidance of COVID-19, and it is the primary channel for people to obtain information [6, 7]. Compared with other network active groups, medical students have higher medical knowledge levels and pay more attention to the relevant reports of the state, government, society, and medical treatment [8–10]. Under the positive reports of medical staff on social media, medical students realize the critical value of the medical profession, which has invisibly affected the formation of their professional identity [11].

Medical students believe that the COVID-19 epidemic will be overcome, which may be an essential factor to encourage them to pay close attention to the progress of epidemic prevention and control, and further affect their attitudes and choices towards the medical profession. To sum up, we hypothesize that the sources of confidence in overcoming COVID-19 can affect the improvement of professional identity through a mediation pathway of attention to COVID-19 among medical students.

**Fig. 1:**
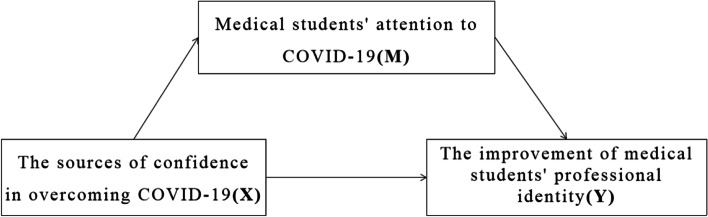
Research hypothesis framework of mediation model

This study aimed to explore the mediation effect of medical students' attention to COVID-19 on the association between the sources of confidence in overcoming COVID-19 and the improvement of medical students' professional identity (See Fig. 1). The findings can provide practical implications for strengthening medical students’ education management theory and practice.

## Methods

### Design

A cross-sectional design was used in this study.

### Setting and participants

The convenience sampling method was adopted to enroll the participants from three universities in the Hunan Province of China from March 15 to April 19, 2020. The study’s inclusion criteria included freshman, sophomore, junior, senior, and fifth-year medical students who volunteered to participate in this web-based survey through the Wenjuanxing platform. The exclusion criterion was withdrawal from the study during the questionnaire completion. A self-report questionnaire was used to collect their basic information, such as age, gender, nationality, university, residence, grade level, and majors. A total of 2960 medical students from three universities were enrolled in the study, and the total response rate was 100%, of which 2775 were valid questionnaires, and the effective rate was 93.8%. All participants provided informed consent before completing the questionnaire, and each participant was paid a total of 7 yuan ($1).

### Sample size

According to the international principles of questionnaire design and previous research experience, the sample size should be 5 to 10 times the number of questionnaire items [12]. A total of 18 items in this study, including seven items on medical students' attention to COVID-19, five items on the improvement of medical students' professional identity, and six items on the sources of confidence in overcoming COVID-19. Consideration of the no reply, incomplete questionnaires, and sample loss, the sample size increased by 20%. Therefore, the required sample size would be 216 and above participants.

### Measurements

#### Medical students' attention to COVID-19

Medical students' attention to national crises was evaluated using seven items: Item 1: “ I am concerned about the progress of COVID-19, such as the trends change of confirmed, suspected, dead, and cured number cases”; Item 2: “I am concerned about the measures taken by the national government to prevent and control the spread of COVID-19”; Item 3: “I am concerned about the measures taken by my local government to prevent and control the spread of COVID-19”; Item 4: “I am concerned about the protection knowledge of COVID-19”; Item 5: “I shared the knowledge of COVID-19 with my friends and relatives during the epidemic period”; Item 6: “I am concerned about people's donation activities to the epidemic areas”; Item 7: “I am concerned about any news or information about the epidemic from the medical staff.” Every item was calculated using a 4-point scale. All subjects could choose the most appropriate item according to their actual situation. The highest score of 4 was “I am concerned about it every day,” and the lowest score of 1 was “I did not pay attention to the news.” The total score of the questionnaire was calculated from the sum of all items. Higher scores indicate that students paid close attention to COVID-19. The research team created a questionnaire according to their professional knowledge, combined with a literature review, expert consultation, and existing research instruments [11, 13–16]. Cronbach's alpha of the scale was 0.921, and the Kaiser–Meyer–Olkin value was 0.913.

#### The improvement of medical students' professional identity

Five items assessed the improvement of medical students' professional identity. Item 1: “The understanding of global epidemic prevention and control through internet media has improved my awareness of health”; Item 2: “The understanding of global epidemic prevention and control through internet media has improved my cognition of scientific and academic rigor of medical knowledge”; Item 3: “The understanding of medical staffs epidemic prevention and control actions through internet media has increased my sense of trust in medical staff”; Item 4: “The understanding of medical staffs epidemic prevention and control actions through internet media has strengthened my respect for the medical profession”; Item 5: “ This public health event has enhanced my sense of identity and employment intention in the medical profession.” Every item was calculated using a 5-point scale ranging from 1 to 5. All subjects could choose the most appropriate item according to their actual situation. The highest score of 5 was “full improvement,” and the lowest score of 1 was “declined a lot.” The total score of the questionnaire was calculated from the sum of all items. The higher the total score, the better the promotion of medical students' professional identity. The questionnaire was created by the research team according to their professional knowledge, combined with a literature review, expert consultation, and existing research instruments [17–22]. Cronbach's alpha of the scale was 0.899, and the Kaiser–Meyer–Olkin value was 0.882.

#### The sources of confidence in overcoming COVID-19

Six items assessed the sources of confidence in overcoming COVID-19. Item 1: “Our country has taken resolute measures to prevent and control the epidemic, which makes me believe that COVID-19 can be overcome”; Item 2: “National leaders attach great importance to epidemic prevention and control, which makes me believe that COVID-19 can be overcome”; Item 3: “The medical staff has provided strong assistance to the epidemic area, which makes me believe that COVID-19 can be overcome”; Item 4: “All over the country, groups have carried out charity donation activities to the epidemic areas, which makes me believe that COVID-19 can be overcome”; Item 5: “The prevention and control measures taken by the local government have gradually brought the epidemic under control, which makes me believe that COVID-19 can be overcome”; Item 6: “The public has gradually understood the knowledge of epidemic prevention and control, which makes me believe that COVID-19 can be overcome.” Students responded “yes” (score 1 point) or “no” (score 0). The total score of the questionnaire was calculated from the sum of all items. All subjects could choose the most appropriate item according to their actual situation. The research team completed the questionnaire according to their professional knowledge, with a literature review, expert consultation, and existing research instruments [23–25]. Cronbach's alpha of the scale was 0.865, and the Kaiser–Meyer–Olkin value was 0.836.

### Statistical analysis

Statistical Package for the Social Sciences software (SPSS, version 26.0) and Excel (Microsoft Corp, Redmond, WA, United States) was used for management and analysis. Descriptive statistics were used to analyze demographic data and all study variables. Spearman correlation analysis was used to analyze the correlation among the study variables. PROCESS Model 4 with 5000 bootstrap samples in SPSS was used to test the mediation model. The bias-corrected bootstrap method was used to estimate the highest statistical efficacy and the most accurate confidence interval [26]. The sources of confidence in overcoming COVID-19 were used as independent variables, medical students' attention to COVID-19 was used as a mediating variable, and the changes in medical students' professional attitudes were used as a dependent variable. The total, direct, and indirect effects were considered statistically significant at the 0.05 probability level if the 95% bias-corrected confidence interval (CI) did not include zero [27].

### Ethical approval

The study was approved by the ethics committees of the University of South China. All participants provided informed consent.

## Results

### Characteristics of the study population

A total of 2960 medical students were enrolled in the study, and 2775 participants responded entirely and reliably to the questionnaire, resulting in a valid response rate of 93.8% (2775/2960). The mean age of the students was 20.80 ± 1.25 years. The general demographic data of the subjects are shown in Table 1. Among the surveyed medical students, 88.1% were female, and 85.2% were of Han nationality. Students' residences were located in cities (24.5%), towns (25.6%), and villages (49.9%), respectively. The average levels of the sources of confidence in overcoming COVID-19 score (0–6), the changes in medical students' professional attitudes score (5–25), and medical students' attention to COVID-19 score (7–28) of respondents were 4.76 ± 1.69, 21.14 ± 4.56, and 21.48 ± 4.21, respectively.

**Table 1:** Demographic characteristics of the study population (*N* = 2775)

Variables	n	Percentage(%)
Gender		
Women	2444	88.1
Men	331	11.9
Nationality		
Han	2363	85.2
Other	412	14.8
Place of residence		
Cities	681	24.5
Towns	710	25.6
Villages	1384	49.9
Grade		
Freshman	2378	85.7
Sophomore	73	2.6
Junior	162	5.8
Senior	128	4.6
Fifth year	34	1.2
Subject category		
Medical humanities	673	24.3
Natural sciences	522	18.8
Biomedical engineering	580	20.9
Medicine	1000	36.0

The subjects of this study were all medical students of medical field. According to subject category, it can be divided into medical humanities (e.g., medical humanities major), natural sciences (e.g., clinical medicine major), biomedical engineering (e.g., biomedical engineering major), and medicine (e.g., pharmacy major)

### Evaluation of the measurement instruments

The construct reliability and validity for the measurement instruments were fair ideals (Table 2). According to Hair et al. [28], the indicator loadings should reach the standard of 0.70. Loadings between 0.40 and 0.70 should be deleted only when their deletion can improve the overall reliability to the minimum threshold value. The results showed that two indicator loadings were lower than 0.70, and the other were higher than 0.70. The overall reliability was not improved by deleting and analyzing the indicator loadings less than 0.70. Therefore, all indicator loadings were retained for this study. The results reflected that the indicator loadings had good indicator reliability levels.

**Table 2:** Evaluation of measurement instruments

**Construct**	**Item**	**Loadings**	**Cronbach’s alpha**	**Composite reliability**	**AVE**^a^	**KMO**^b^
The sources of confidence in overcoming COVID-19 (SCOC)	SCOC-1	0.706	0.865	0.860	0.508	0.836
	SCOC-2	0.775				
	SCOC-3	0.770				
	SCOC-4	0.734				
	SCOC-5	0.639				
	SCOC-6	0.639				
The improvement of medical students' professional identity (IMSPI)	IMSPI-1	0.801	0.899	0.932	0.732	0.882
	IMSPI-2	0.844				
	IMSPI-3	0.883				
	IMSPI-4	0.883				
	IMSPI-5	0.865				
Medical students' attention to COVID-19 (MSAC)	MSAC-1	0.822	0.921	0.939	0.687	0.913
	MSAC-2	0.886				
	MSAC-3	0.892				
	MSAC-4	0.883				
	MSAC-5	0.754				
	MSAC-6	0.727				
	MSAC-7	0.823				

^a^Average Variance Extracted

^b^Kaiser-Meyer-Olkin

### Spearman correlation analysis results

A nonparametric statistical method was used for bivariate correlation. Table 3 shows the overall Spearman correlation results. There were significant correlations between all variables. The improvement of medical students' professional identity was positively correlated with the sources of confidence in overcoming COVID-19 (*r* = 0.629, *P* < 0.001) and medical students' attention to national crisis events (*r* = 0.791, *P* < 0.001).

**Table 3:** Spearman correlations among study variables

Variables	1	2	3
1. The sources of confidence in overcoming COVID-19	1		
The improvement of medical students' professional identity	0.629^**^	1	
Medical students' attention to COVID-19	0.617^**^	0.791^**^	1
Mean	4.76	21.14	21.48
SD	1.69	4.56	4.21

*N* = 2775

^**^All correlations were significant at *P* < 0 .001 (two tailed)

### Mediation effect model

**Fig. 2:**
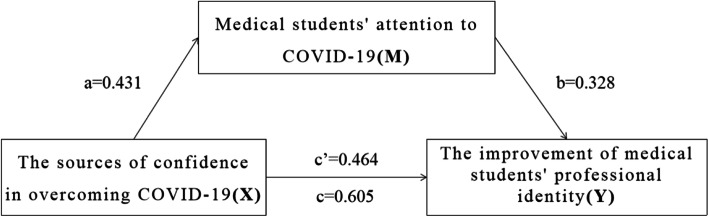
Mediation model of how does the sources of confidence in overcoming COVID-19 influences the improvement of medical students' professional identity via medical students' attention to COVID-19. a = direct effect of X on mediator M; b = direct effect of mediator M on Y; c = total effect of X on Y; c’ = direct effect of X on Y

Medical students' attention to COVID-19 was identified as a mediator between the sources of confidence in overcoming COVID-19 and the improvement of medical students' professional identity. In the SPSS PROCESS analysis, model 4 was used to evaluate the mediating effects of the study variables. The total, direct and indirect effect results are summarized in Table 4. Figure 2 shows the structural model of the mediating effect. The structural model was statistically significant (*F* = 223.495, *P* < 0.001), and the *R* and *R*^*2*^ values were 0.373 and 0.139, respectively (Table 5). The total effect of the mediation model was significant. The direct effect of the sources of confidence in overcoming COVID-19 on the improvement of medical students' professional identity was significant. The indirect effect of the sources of confidence in overcoming COVID-19 (X) on the improvement of medical students' professional identity (Y) through medical students' attention to COVID-19 (M) was significant. The mediation effect accounted for 23.3% of the total effect. The mediation effect accounted for 30.4% of the direct effect. Therefore, these research results indicate that a partial mediating effect exists.

**Table 4:** Total, direct, and indirect effects of mediation analysis

Effect	Estimate	*t/Z* value	*P* value	95% LCL	95% UCL
Total effect	0.605	12.101	< 0.001	0.507	0.703
Direct effect	0.464	9.597	< 0.001	0.369	0.559
Indirect effect	0.141	8.092^a^	< 0.001	0.106	0.182

^a^*Z* value

**Table 5:** Regression model with the improvement of medical students' professional identity as outcome variable

Variables	coeff	se	*t* value	*P* value	LLCI	ULCI
Constant	11.892	0.447	26.591	< 0.001	11.015	12.769
Medical students' attention to COVID-19	0.328	0.019	16.898	< 0.001	0.290	0.366
The sources of confidence in overcoming COVID-19	0.464	0.048	9.597	< 0.001	0.369	0.559

## Discussion

This study first explored the role of medical students' attention to COVID-19 on the sources of confidence in overcoming COVID-19 and the improvement of medical students' professional identity. We developed a mediation model to assess their direct and indirect relationships. We found that the sources of confidence in overcoming COVID-19 are positively associated with the improvement of medical students' professional identity. Medical students' attention to COVID-19 plays a partial medium role between them. These findings are essential additions to existing research on the theory and practice of professional identity among medical students.

### Implications for theory

This study found that the sources of confidence in overcoming COVID-19 play an essential role in improving medical students’ professional identity. The emergency management of COVID-19 requires full participation, mobilizing the forces of individuals, families, communities, social organizations and other aspects, forming an environmental atmosphere for the public to fight against the epidemic. The expected positive emotional experience and mutual social trust make the public firmly believe that the risk of public health events will be overcome [21]. The government, medical workers, scientific researchers, and grass-roots organizations are the leading players involved in preventing and controlling the COVID-19 epidemic. Their achievements are the source of public confidence that the epidemic will be overcome. Studies show that 43.3%, 46.3%, 53.6%, and 42.7% of the public have confidence in scientific researchers, medical staff, government, and grass-roots to overcome COVID-19, respectively [29]. In particular, medical staff members work day and night on the medical front and have established a high sense of professional responsibility and morality for medical students, which is conducive to enhancing medical students' medical professional identity [30].

The results of the current study demonstrated that medical students' attention to COVID-19 plays a partially medium role in the sources of confidence in overcoming COVID-19 and the improvement of professional identity. Social media is the primary channel for the public to pay attention to preventing and controlling COVID-19. According to statistics, WeChat (82.4%) and Twitter (75.8%) were the main channels, followed by television (42.9%), websites (41.1%), and news clients (40.8%) [29]. The network media concentrated on reporting the effectiveness of joint prevention and control measures of various regions and departments have strengthened the confidence of medical students that the country and its people will eventually overcome the virus and further encouraged them to continue to pay attention to the development of national COVID-19. They are interested in a wealth of information about COVID-19, such as disease development, treatment methods, preventive measures, vaccine research, and medical team dispatch [4, 7, 8]. At the same time, medical students are expected to apply their knowledge to prevention and control work. A study found that approximately 83.85% of medical students were moved by the behavior exhibited by front-line medical staff, who devoted much of their time and energy to work [31]. About 85.7% of medical students are willing to take the initiative to participate in front-line work in the future [32]. It can be seen that medical students are full of confidence to overcome the COVID-19 epidemic, which is conducive to promoting their continued to pay attention to the progress of COVID-19 prevention and control and further improve their professional identity.

Despite the high social status of medical staff, the work nature of high-risk, high-pressure, and long working hours leads to some medical students’ low professional identity scores [17, 33]. In the progress of prevention and control of COVID-19, the strong support of social groups for medical work and the high frequency and intensity of positive reports by the news media have greatly improved the professional identity of medical students, which has deeply enhanced their sense of responsibility and mission [11]. Studies have shown that 59.0% of medical students are willing to participate in front-line epidemic prevention actions, and 87.7% of medical students have experienced a positive impact on their professional identity [33]. Other studies have shown that 42.9% of medical students have not been shaken in their professional beliefs during the COVID-19 epidemic, and 53.6% of medical students have strengthened their beliefs [34]. Therefore, the support of the government, society, and media to medical staff is essential to improve medical students’ professional identity.

### Implications for practice

Improving medical students' professional identity is a critical target of professional identity education and an essential task of national development and social stability. According to our study results, we propose the following recommendations.

First, community practice can provide a theoretical basis for professional identity [35, 36]. During COVID-19, the anti-epidemic activities of medical staff were the most regular, large-scale community practices in the epidemic areas. Colleges should pay attention to the role of community practice courses in shaping professional identity. Medical students could establish their professional image through experiential learning in the Centers for Disease Control and Prevention (CDC), Community Health Centers, clinical hospitals, and other organizations [37]. In the interactive process of social practice, they can think, act and feel like medical staff, and gradually understand the essence of professional identity and the socialization process of identity formation [35]. Some scholars believe that the most influential factors influencing professional identity formation are role models and mentors, and experiential learning in clinical and nonclinical situations [38, 39]. In community practice, students consciously acquire knowledge through observation, imitation, and practice [33, 40, 41]. Role models and mentors can encourage medical students to reflect and summarize in practical learning to enhance their learning effect considerably [33]. These measures enable medical students to avoid overreliance on historical precedent, intuition, or perceived common sense and improve their underlying public health service skills. In addition, medical colleges need to set up special practical skills courses on how to deal with the doctor-patient relationship to improve medical students' medical confidence and thus enhance their willingness to practice medicine [42].

Second, social media is essential for improving the cognitive domain [43]. The attention of medical students to media reports reflects the critical role of social media in the construction of medical students' values and professional sense to a certain extent. Medical students pay close attention to the progress of public health events through social media. They discuss issues with peers online, give feedback and show their attitude, and are influenced by each other through the media [10]. Positive reports of COVID-19 can improve medical students' confidence in overcoming COVID-19. However, relevant information about COVID-19 is often exaggerated in the spread of social media. Even those not directly affected will form a particular position or attitude, causing forwarding, commenting, and other behaviors, further promoting the spread and diffusion of public opinion information on social media platforms. This situation can spread anxiety, panic, and other negative emotions, thus shaking confidence in overcoming crisis events [44]. At the same time, the adverse reports on doctor-patient disputes by the news media have made medical students’ levels of cognition and emotion toward medical work more one-sided [45]. From this, government departments should first take a clear stand on public opinion during the crisis, reasonably guide public opinion, and positively build public confidence in dealing with COVID-19. Then, they need to monitor and analyze the public opinion information of public health emergencies, guide medical students to understand the medical and practice environments objectively, and clarify the professional situation of medical staff. The media should report the significance of medical personnel in dealing with COVID-19, which is conducive to stabilizing the supply of human medical resources and improving the quality of medical services. Additionally, the idea of modern medical education is to guide medical students to make full use of network media in a positive way to improve their professional identify to promote the transformation of their self-awareness from university medical students to future healthcare professionals [46]. Consequently, the correct levels of guidance from the government and the media allow medical students to choose their jobs objectively and strengthen their professional beliefs when paying attention to COVID-19.

Finally, society and family support are essential factors for medical students to strengthen their professional consciousness. During COVID-19, the sources of confidence in overcoming COVID-19 from the government and society reflected the most vital social support. In addition to professional courses, employment guidance and training, society and family are important factors affecting medical students' professional identity. Many nurses think that patients do not respect nursing work and that nurses have a lower social status. When the professional status of medical personnel is low, dismissal may occur, especially for male nurses who suffer from gender stereotypes and lack social and family support [47, 48]. It is well known that nursing staff are an indispensable part of medical activities, especially since the role of male nurses is becoming increasingly prominent. These warn the national relevant departments, such as holding regulators, hospitals, and nursing educational institutions, to develop the professional identity of male nurses, emphasizing personalized training and guidance, such as lectures, competitions, or salons, to realize their social value fully. Participation in these activities can change their perception of the nursing profession and help maintain the stability of male caregivers [49, 50]. In addition, family conflict was negatively correlated with professional identity. Faced with the current heavy academic burden and high social requirements of medical students, families should create a healthy and relaxed environment. When the country in a significant public health crisis, families should actively support medical students to participate in primary healthcare workers and encourage them to promote their professional competence and vocational awareness.

### Strengths, limitations, and future research

The study makes an essential contribution to the literature by evaluating the associations among the sources of confidence in overcoming COVID-19, the improvement of medical students' professional identity, and medical students' attention to COVID-19 through data reported by medical students from three medical universities in Hunan Province. The present study confirms our research hypothesis that medical students' attention to national crisis events moderates the relationship between the sources of confidence in overcoming COVID-19 and the improvement of medical students' professional identity. Additionally, these findings enrich the theory and practice of professional identity education-related research among medical students.

Our study has several limitations. First, our study is a cross-sectional study, which can only account for the associations between these three and cannot explain the causal relationships between them. Second, this study was based on the self-reported of students to assess the improvement of their professional identity, which may have led to a response bias. Third, medical students' professional attitudes result from many factors, and we explored only their attention to COVID-19 as an intermediary factor. In future research, longitudinal research is needed to confirm these contentions. The predictive effect of other personal factors, such as medical students' participation in epidemic prevention and control workers or social and family factors on medical students' professional identity, need to be further studied. Beyond that, their association and role in the context of other public health events requires further research.

## Conclusions

In conclusion, this study confirms the importance of the association among the sources of confidence in overcoming COVID-19, the improvement of medical students' professional identity, and medical students' attention to COVID-19. The sources of confidence in overcoming COVID-19 and medical students' attention to national crisis events have a significant predictive effect on improving medical students' professional identity. Medical students' attention to COVID-19 mediated the relationship between the sources of confidence to overcome COVID-19 and the improvement of medical students' professional identity. These findings suggest that enhancing confidence in overcoming COVID-19 and medical students' attention to COVID-19 can meaningfully improve medical students' professional identity. Our findings will serve as a springboard for future research on improving medical students' professional identity.

## Data Availability

There is no public availability to the interview transcripts outside of the research team due to reasons of confidentiality. Data are however available from the corresponding author on reasonable request.
